# PrionW: a server to identify proteins containing glutamine/asparagine rich prion-like domains and their amyloid cores

**DOI:** 10.1093/nar/gkv490

**Published:** 2015-05-14

**Authors:** Rafael Zambrano, Oscar Conchillo-Sole, Valentin Iglesias, Ricard Illa, Frederic Rousseau, Joost Schymkowitz, Raimon Sabate, Xavier Daura, Salvador Ventura

**Affiliations:** 1 Institut de Biotecnologia i de Biomedicina and Departament de Bioquímica i Biologia Molecular, Universitat Autònoma de Barcelona, Bellaterra, 08193, Spain; 2 VIB Switch Laboratory and Department for Cellular and Molecular Medicine, KU Leuven, Leuven, Belgium; 3 Institut de Nanociència i Nanotecnologia (IN^2^UB) and Departament de Fisicoquímica, Facultat de Farmàcia, Universitat de Barcelona, Barcelona, Spain; 4 Institució Catalana de Recerca i Estudis Avançats (ICREA), Barcelona, Spain

## Abstract

Prions are a particular type of amyloids with the ability to self-perpetuate and propagate *in vivo*. Prion-like conversion underlies important biological processes but is also connected to human disease. Yeast prions are the best understood transmissible amyloids. In these proteins, prion formation from an initially soluble state involves a structural conversion, driven, in many cases, by specific domains enriched in glutamine/asparagine (Q/N) residues. Importantly, domains sharing this compositional bias are also present in the proteomes of higher organisms, thus suggesting that prion-like conversion might be an evolutionary conserved mechanism. We have recently shown that the identification and evaluation of the potency of amyloid nucleating sequences in putative prion domains allows discrimination of genuine prions. PrionW is a web application that exploits this principle to scan sequences in order to identify proteins containing Q/N enriched prion-like domains (PrLDs) in large datasets. When used to scan the complete yeast proteome, PrionW identifies previously experimentally validated prions with high accuracy. Users can analyze up to 10 000 sequences at a time, PrLD-containing proteins are identified and their putative PrLDs and amyloid nucleating cores visualized and scored. The output files can be downloaded for further analysis. PrionW server can be accessed at http://bioinf.uab.cat/prionw/.

## INTRODUCTION

Prions are a class of proteins that can exist in at least two conformations, one of which is an amyloid state that is self-propagating and hence infectious as it can induce the conversion of identical protein sequences from the nonprion conformation to the amyloid state (1). Although prions were discovered through the example of the human pathogen PrP (2), a host of functional prions have since been discovered, predominantly in fungi (3,4). Importantly, the distinction between prion proteins and other proteins capable of forming amyloids is blurring, notably in human diseases such as Alzheimer's or Parkinson's, as it has been observed that amyloids of the proteins involved in these diseases are capable of seeding amyloid formation of the soluble form of these proteins, both *in vitro* and *in vivo* laboratory conditions (5,6). Given that there is no epidemiological evidence that these amyloidogenic proteins are spreading in natural systems, the group has been called prion-like or ‘prionoid’ (1). This raises the question of what sequence determinants characterize a functional prion beyond mere amyloid propensity. A subset of prions, not including PrP, are multidomain proteins containing both globular domains and, usually, one Prion Forming Domain (PFD) enriched in glutamine and asparagine (Q/N) residues that undergoes the structural rearrangement during prion conversion (7). Most known yeast prions, but not all, share this architecture. The sequence features of these PFDs overlap with those of intrinsically disordered regions (DRs) (8). It has been proposed that in contrast to the short stretches that are known to be sufficient to nucleate amyloid formation, Q/N based yeast prions have more diffuse nuclei, characterized by a large number of weak interactions between the side-chains of the PFD (9,10). However, we have demonstrated that the superimposition of an intrinsically disordered sequence region containing ‘classical’ amyloid nucleating sequences in fact yields a more accurate classification of experimental prions from related Q/N-enriched sequences (11). In the current paper, we provide public access to our method by way of a webserver.

## METHOD

PrionW allows scanning individual protein sequences for the presence of putative Q/N rich PFDs, as well as the scanning of large protein datasets (up to 10 000 sequences) for proteomic analysis. The method behind PrionW assumes that in order to be a PFD a protein sequence should fulfill the following requirements: (i) contain a specific stretch with significant amyloid propensity, able to selectively nucleate self-assembly into ordered, but brittle, amyloid structures, (ii) have a disordered structural context that readily permits self-assembly without requiring conformational unfolding and (iii) have an amino acid composition that allows the domain to be soluble at the physiological concentrations required for protein function yet display a basal amyloid propensity, to which N and Q residues would contribute significantly, promoting domain assembly in the presence of preformed amyloid seeds or when the concentration is increased.

**Figure 1.**
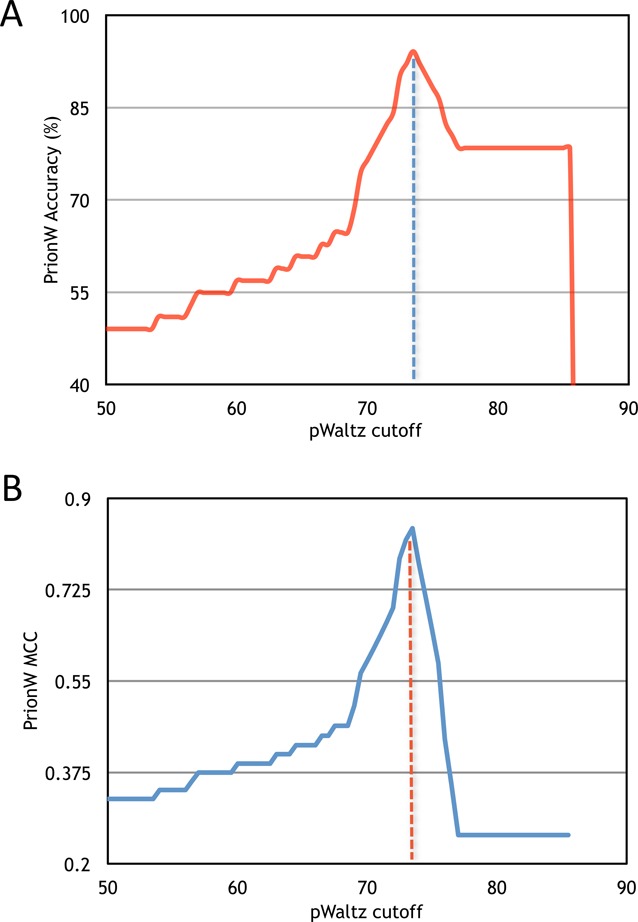
Accuracy and MCC cut-off plot for PrionW. (**A**) Accuracy, in percentage, and (**B**) Matthews correlation coefficient (MCC) obtained for the correct classification of TP and TN is graphed against increasing pWALTZ cut-offs. We highlight the highest accuracy and MCC of the assay, used to set the predictive cut-off of 73.55.

PrionW analyses whether a given protein or protein fragment satisfies the above requirements in three sequential steps:

- Identification of DRs in protein sequences: PrionW analyzes protein sequences to identify the presence of intrinsically DRs by implementing FoldIndex (12) with the default 51-aa window size. Only disordered segments of at least 60 contiguous residues are further evaluated, since this core size seems to suffice to attain a prion-like behavior in Q/N rich PFDs (13). When a protein contains two or more DRs, these regions are subsequently evaluated individually.
- Evaluation of Q/N enrichment: The proportion of Q + N residues in the detected DRs is calculated. The program moves through each individual sequence by single amino acid steps looking for the longer stretch of adjacent residues having a Q/N proportion ≥ than a given threshold in the predicted disordered region. The default is set at ≥ 25% of Q/N residues, because the PFDs of most experimentally characterized yeast prions fulfill this requirement (13,14). However, since Q/N enrichment for prion-like formation might change from organism to organism the user can select the minimum Q/N content. If the threshold is set to 0% the program identifies only DRs.
- Amyloid core identification and scoring: The individual sequences fulfilling the requirements in steps 1 and 2 are further evaluated for the presence of a 21-residue long amyloid core able to specifically nucleate its self-assembly according to the pWALTZ scoring function (11), an update of the scoring function in our well-established amyloid predictor WALTZ (15). The default pWALTZ cut-off in PrionW was set to 73.55, since this value provided the best accuracy for the discrimination of experimentally validated putative yeast PFDs from sequences displaying similar Q/N content but devoid of prionogenic potential (see Performance). A lower cut-off can be useful to identify sequences in genomes with a basal prion propensity (16). Accordingly, the user can select the pWALTZ cut-off in the 50–75 value range. pWALTZ values lower than 50.0 are not allowed because they do not permit discrimination of prion and non prion sequences in the yeast dataset used for parameterization, since the accuracy of PrionW in these conditions is below 50% and the Matthews correlation coefficient (MCC) below 0.4 (Figure 1). For a given protein sequence, the disordered Q/N rich region containing the highest-scoring amyloid core is selected as the putative PFD or Prion-like domain (PrLD) in this protein, as long as it passes the selected threshold.

## PERFORMANCE

Yeast prions constitute ideal model systems to characterize prion-like behavior. On the basis of compositional similarity to known prions, Lindquist's group used a hidden Markov model (HMM) to identify 100 prion candidates in the yeast genome (13). They scored 92 of them from 0 to 10 according to their performance in four different experimental assays for both amyloid and prion forming ability, higher scores indicating more prionogenic sequences. It turned out that in this, in principle, prion enriched set, only 13% of the proteins scored ≥ 9 whereas 42% scored ≤ 2, demonstrating the extreme difficulty to discriminate prions from nonprions when they all share a similar Q/N enriched compositional context. The HMM predicted PFDs of these proteins were used to build up a dataset in which we considered as nonprions (negatives) those sequences scoring ≤ 2 and being positive in one assay at maximum (39 sequences), because it means that they do not exhibit amyloid and prion forming ability at the same time, and prions (positives) those domains being positive in all four assays and scoring ≥ 9, with a total of 12 sequences, including the actual prions New1, Rnq1, Swi1, Sup35 and Ure2 proteins, defined as those proteins that act as prions in its natural context, increasing population-level phenotypic heterogeneity (11) (Supplementary Table S1). We speculated that the presence and the strength of short amyloid cores embedded in these domains might account for their different prionogenic potential. This concept was implemented in the pWALTZ scoring function, allowing discrimination between positive and negative PFDs in the above-mentioned 51-protein dataset with better accuracy than approaches based only on composition (11).

Despite its accuracy, a serious limitation of pWALTZ to analyze large protein datasets is that it needs to work on top of dissected putative PFDs sequences, because the folded domains adjacent to these regions and, more generally, globular proteins usually contain one or more amyloid regions (17), whose high aggregation potency would blur any prediction. PrionW tries to solve this issue by considering the structural disorder and Q/N compositional bias characteristic of most yeast PFDs.

In our previous work, a 73.55 pWALTZ cut-off provided the best accuracy to discriminate prions from nonprions (11); however, this value resulted from the analysis of the PFDs identified by the Lindquist's group HMM, which may or may not coincide with those sequences identified by PrionW on the basis of structural disorder and Q/N content for their further pWALTZ assessment (see Methods). Thus, to parameterize PrionW, we analyzed the 6719 proteins encoded in the *Saccharomyces cerevisiae* S288c reference proteome for the presence of PrLDs using a fixed Q/N content ≥ 25% and gradually increasing the pWALTZ cut-off from 35 to 90% in 0.1% steps. The accuracy of the method was calculated at each stringency level by evaluating the presence of positive and negative instances from the original 51-protein dataset (Supplementary Table S1) in the returned proteome predictions (Figure 1A). The best predictions were obtained with cut-offs ranging from 73.50 to 73.60, suggesting that the disordered Q/N rich domains identified by PrionW might overlap with the PFDs identified using the HMM. We also calculated the MCC associated at the prediction at each stringency level (Figure 1B). Again the best correlations were obtained in the 73.50–73.60 range. Accordingly, a 73.55 cut-off was selected as the default pWALTZ value in PrionW. The performance of the predictions is maintained at any Q + N richness in the ≥ 20% to ≥ 35% range. Using a Q/N content ≥ 25% and a 73.55 pWALTZ cut-off, PrionW returned a total of 63 predictions (Supplementary Table S2). They included 92% of the previously considered positives (11 sequences), only Puf2 being missing. In contrast, only 5% of the negative ones (two sequences) were recovered. This corresponds to a sensitivity of 0.917, a specificity of 0.949, a precision of 0.846, an accuracy of 0.941 and a false discovery rate of only 0.154. These values (Table 1) indicate that our methodology produce fairly clean recovery sets with a rather low proportion of false positives. If we consider as positive sequences only the set of actual Q/N-rich prions: Cyc8, Mot3, New1, Rnq1, Sfp1, Swi1, Sup35 and Ure2, PrionW is able to recover the large majority of them from the yeast proteome with the default settings, missing only Cyc8.

**Table 1.** Performance of DIANA, LPSs and PrionW approaches in the prediction of experimental yeast prion-like proteins (protein dataset in Supplementary Table S1)

	DIANA	LPSs	PrionW
Sensitivity	0.917	**1**	0.917
Specificity	0.385	0.128	**0.949**
Precision	0.314	0.261	**0.846**
FDR^a^	0.686	0.739	**0.154**
Accuracy	0.510	0.333	**0.941**
MCC^b^	0.275	0.183	**0.842**

^a^False Discovery Rate.

^b^Matthews correlation coefficient.

The best value for each parameter is indicated in bold.

Two pioneering works addressed previously the discovery of potential novel prion-forming proteins exploiting their Q/N bias. Michelitsch and Weissman developed DIANA (Defined Interval Amino acid Numerating Algorithm), an algorithm aimed to identify proteins containing regions of consecutive amino acids with exceptionally high Q/N content (18). The screening of the yeast proteome with this approach rendered a total of 107 predictions, which include 11 of the positive sequences but also 24 of the negative ones (Supplementary Table S3). Thirty three proteins predicted by PrionW are also present in the DIANA dataset (Supplementary Table S4). Harrison and Gerstein derived a method for identifying biased regions that relies on defining the lowest-probability subsequences (LPSs) for a given amino-acid composition and applied this formalism to analyze the prevalence of Q- and N-rich regions in different proteomes (19). This method identifies 172 prion-like Q/N-rich regions in yeast, which include all the 12 positive sequences but also 34 of the negative ones (Supplementary Table S5). Thirty five proteins predicted by PrionW are also present in the LPS dataset (Supplementary Table S6). A comparison of the performance of PrionW, with that of the DIANA and LPSs approaches (Table 1), illustrates the usefulness of evaluating the presence and potency of short amyloidogenic regions in the context of Q/N rich sequences to discriminate prionogenic sequences in complete proteomes.

The ability to perform predictions in complete proteomes allows using Gene Ontology (GO) annotations to classify proteins containing PrLDs according cellular locations, functional classes and processes, uncovering the role played by these polypeptides in the cell. According to the GO classification in the Sacharomyces Genome Database (SGD) (http://www.yeastgenome.org) the detected proteins are associated to cytoplasmatic ribonucleoprotein granules (*P* = 4.1 × 10^−05^) and nucleus (*P* = 6.1 × 10^−05^), their preferential function is mRNA binding (*P* = 3.0 × 10^−05^) and more generally nucleic acid binding (*P* = 6.3 × 10^−03^) and they work in the regulation of biological processes (*P* = 5.9 × 10^−07^) and more specifically in the regulation of gene expression (*P* = 7.7 × 10^−06^). This analysis highlight the important role played by PrLDs-containing proteins in the yeast physiology, a role that might be also exerted in higher organisms.

**Figure 2.**
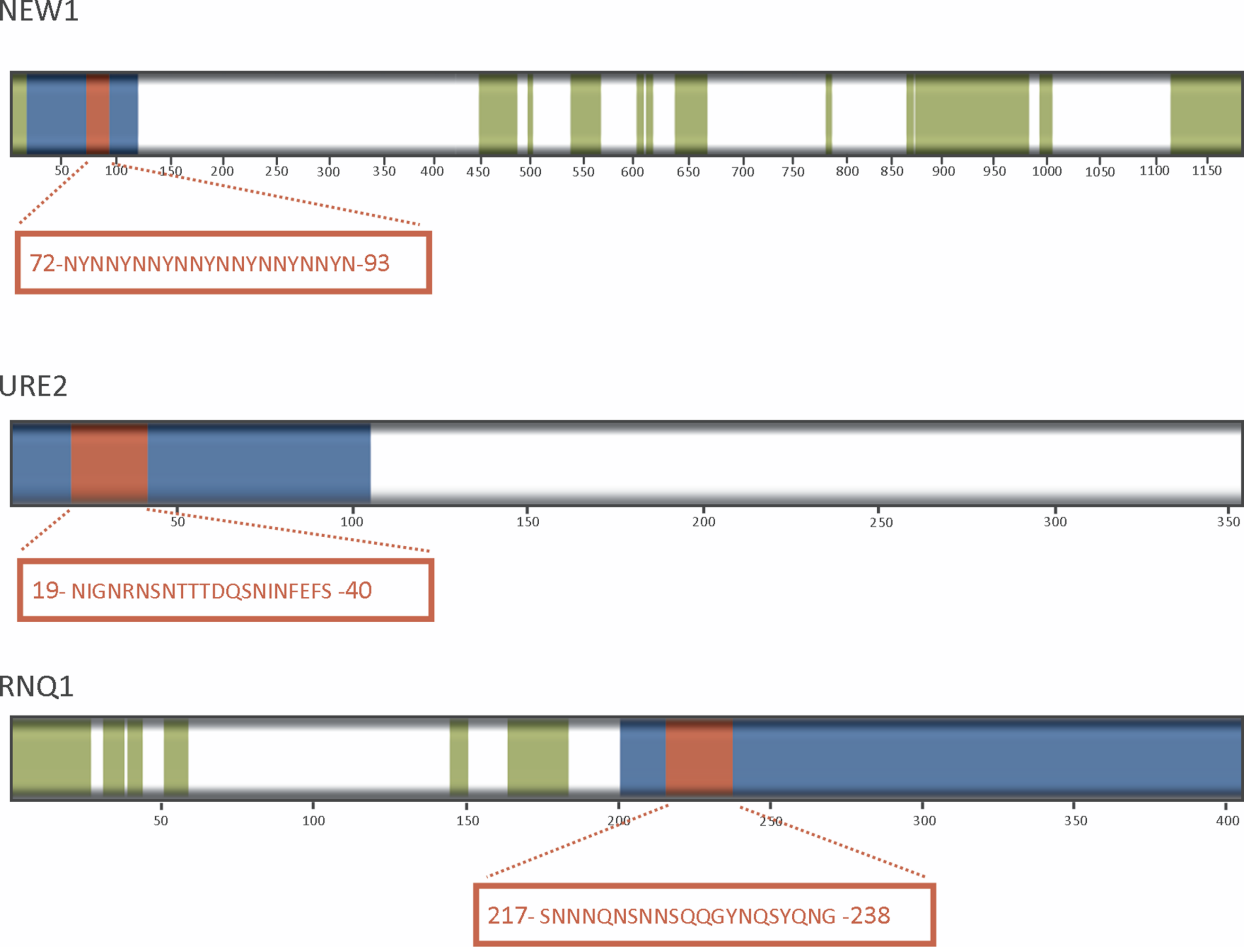
PrionW predictions of prion-like domains (PrLDs) and amyloid cores in the sequences of the genuine yeast prions New1, Ure2 and Rnq1. Folded domains, disordered regions, predicted PrLDs and amyloid cores are shown in white, green, blue and red, respectively.

According to FoldIndex and other disorder predictors like RONN (20) or FoldUnfold (21), in most of the 62 hits, the detected PrLDs are accompanied by at least a folded domain, which is likely the responsible of the protein activity and probably widely offset from the fibril backbone in the amyloid state (22). As expected, in contrast to pWALTZ, PrionW can identify genuine prions even when their PFDs represent a small fraction in the complete sequence of an essentially folded protein (Figure 2). PrionW is not intended to delimit the exact boundaries in the identified PrLDs; however, the best overlap between Uniprot annotated prion domains for actual yeast prions and PrionW predictions was obtained when Q + N richness was set to ≥ 32% (Supplementary Table S2).

The requirement to tune the Q + N content and pWALTZ parameters when using PrionW to screen for prion-like proteins in proteomes different from that of yeast is best illustrated by the fact that the algorithm is not able to identify a set of human proteins that have been proposed to display prion-like behavior (8), including hnRNPA1, hnRNPA2, hnRNPA3, hnRNPDL, SS18L1/CREST, FUS, EWS, TAF15 and TPD43 with the default settings. However, setting the Q/N content at ≥15% and pWALTZ cut-off at 64.00 allows retrieving them, except TDP43, and identifying their putative amyloid cores (Supplementary Table S7). The overall lower amyloidogenic potential of the predicted nucleating cores of those human prion-like proteins could respond to the fact they might not be actual prions, but rather proteins able to self-assemble reversibly for functional purposes (8) and, even if they have been shown to form intracellular aggregates upon mutation (16), their assemblies are generally softer than those of actual yeast prions (23) and it is not evident that they can be propagated as *bona fide* prions.

**Figure 3.**
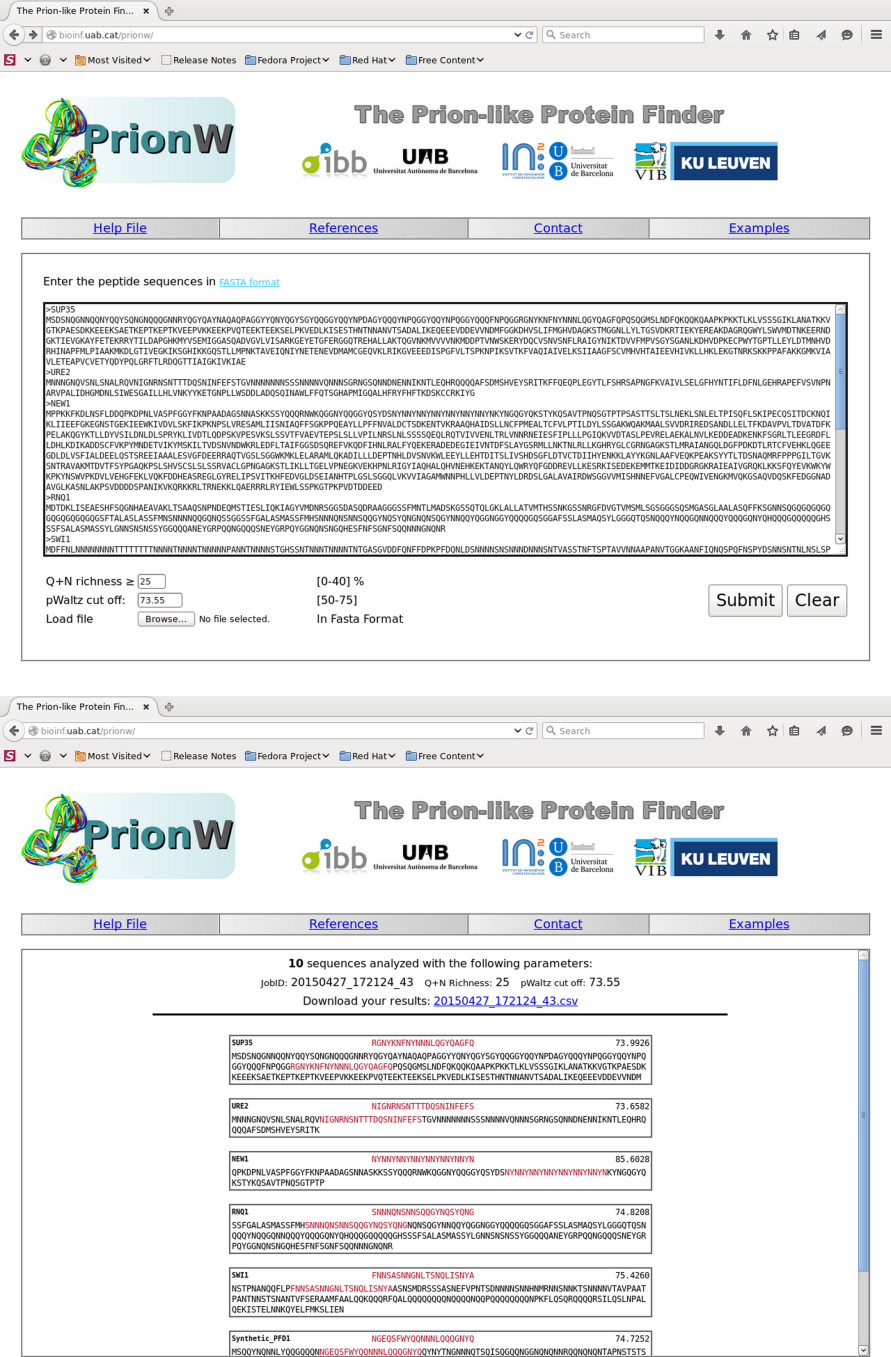
Screen shots of the PrionW web server. Upper panel shows web page input populated with example sequences and algorithm parameters in default values. Lower panel shows the returned results for these sequences.

## SERVER DESCRIPTION

The PrionW webserver does not require any user registration or identification. The interface can process up to 10 000 sequences at a time.

### Input interface

PrionW is presented as an application running in a single web page (Figure 3). One or more sequences in FASTA format must be pasted in the text box or uploaded as a file. Two algorithm parameters can be tuned by the user: ‘Q + N richness’ defines the minimum proportion of Q and N residues a disordered region should have to be further considered; ‘pWaltz cut off’ defines the minimum pWaltz score for an amyloid core to be considered positive. Default values are otherwise assigned to these parameters (see methods for more details). The web page displays four links in its upper margin: (i) reference publications of methods and web application, (ii) a contact mail, (iii) a help with a short description of the algorithm, input instructions, output explanation and information on examples and (iv) examples that will populate the input text area with full-length sequences of the well-characterized yeast prions New1, Rnq1, Swi1, Sup35 and Ure2 and a set of prion positive and negative control synthetic sequences proposed by Toombs *et al*. (10).

### Output

When clicking the submit button the input frame changes. After checking for the correct FASTA format, a header showing the number of interpreted sequences, input parameters and job identifier (to be used in questions to the authors) appears. After the calculation has finished, a link to a CSV file containing the output data are provided. Below the header, positive hits are printed in the same order as they were submitted. For each hit, the name, the predicted 21-residue amyloid core, the pWaltz score and the predicted Q/N rich PrLD with the amyloid core highlighted in red are presented (Figure 3). If no positive sequences are detected in the input dataset the algorithm returns the following message: ‘None of your sequences contains a predicted Prion-like Domain.’ The CSV file contains all the mentioned information, which will be kept in our server for 7 days. If the algorithm detects a format error in the input sequences, an input parameter out of range, a number of sequences exceeding the maximum allowed or an engine problem, the program execution stops and a brief explanation of the problem is given.

### Implementation and server run-time

The PrionW web server runs on an engine implementing the algorithm described in methods. It has been written in python and uses python2.7 as the interpreter. The web interface has been build using html/css and inputs and outputs are processed by a cgi written in perl. It all runs in a CentOS 5 server with Apache 2.2.3 using Intel Xeon ‘Clovertown’ processors. The execution time for processing the complete *S. cerevisiae* S288c reference proteome in our server was lower than 5 min.

## CONCLUSION

We have described PrionW, a web server for the prediction of proteins containing Q/N rich prion-like domains and their amyloid cores in large sequence datasets. The algorithm should find application in the discovery of new candidates in different organisms for further experimental characterization, in the identification of mutations endorsing wild-type proteins with increased prion-like properties, in the design of synthetic prion domains for different purposes or in the design and synthesis of short peptides corresponding to PrLDs amyloid cores able to seed the aggregation of the complete protein and, more generally, in understanding prion function and regulation in different species.

## SUPPLEMENTARY DATA

Supplementary Data are available at NAR Online.

SUPPLEMENTARY DATA
