# Supplementary material for: PrionW: a server to identify proteins containing glutamine/asparagine rich prion-like domains and their amyloid cores
Source: Nucleic Acids Res. 2015 May 14;43(Web Server issue):W331–7. doi: 10.1093/nar/gkv490 (PMC4489250; doi:10.1093/nar/gkv490)
Supplement: SUPPLEMENTARY DATA [file supp_43_W1_W331__index.html]

PrionW: a server to identify proteins containing glutamine/asparagine rich prion-like domains and their amyloid cores — SUPPLEMENTARY DATA 

# PrionW: a server to identify proteins containing glutamine/asparagine rich prion-like domains and their amyloid cores

## SUPPLEMENTARY DATA

- SUPPLEMENTARY DATA
