# Supplementary material for: PrionW: a server to identify proteins containing glutamine/asparagine rich prion-like domains and their amyloid cores
Source: Nucleic Acids Res. 2015 May 14;43(Web Server issue):W331–7. doi: 10.1093/nar/gkv490 (PMC4489250; doi:10.1093/nar/gkv490)
Supplement: SUPPLEMENTARY DATA [file supp_gkv490_nar-00488-web-b-2015-File005.pdf]

SUPPLEMENTARY DATA

METHODS

Calculation of algorithms performance:

Sensitivity or true positive rate was calculated as: Sensitivity = TP/P = TP/(TP+FN).

Specificity or true negative rate was calculated as: Specificity = TN/N = TN/(FP+TN).

Precision or positive predictive value was calculated as: Precision = TP/(TP+FP)

Accuracy was calculated as: Accuracy = (TP+TN)/(P+N)

False discovery rate (FDR) was calculated as: FDR = FP/(TP+FP)

Matthews correlation coefficient (MCC) was calculated as:

MCC = (TPxTN – FPxFN) / √((TP+FP)x(TP+FN)x(TN+FP)x(TN+FN))

Where P = Positives, N = Negatives, TP = True Positives, FP = False Positives, TN = True Negatives and FN = False Negatives.

Table S1. Dataset of the 51 proteins containing the putative PFDs used in the benchmarking of pWALTZ (1). Prion/Non-Prion (P/NP) classification according to Alberti *et. al.* scale of prion propensity (2). Sequences scoring ≤ 2 (1 positive assay as a maximum) were considered non-prions (NP) while sequences scoring ≥ 9 (all four assays positives) were considered prions (P). PFDs were as described in (2).

Uniprot Entry	Gene Name	Prion/Non Prion	Uniprot Entry	Gene Name	Prion/Non Prion
Q12221	PUF2	P	Q00772	SLT2	NP
P09547	SWI1	P	P41696	AZF1	NP
P38691	KSP1	P	P31384	CCR4	NP
Q05166	ASM4	P	P48837	NUP57	NP
P23202	URE2	P	P24276	SSD1	NP
P18494	GLN3	P	Q08831	VTS1	NP
P25367	RNQ1	P	P50896	PSP1	NP
Q08972	NEW1	P	P53309	YAP1802	NP
P32770	NRP1	P	P39523	YMR124W	NP
P40070	LSM4	P	Q05785	ENT2	NP
P38180	YBL081W	P	P32900	SKG6	NP
P05453	SUP35	P	Q06251	YLR177W	NP
P11746	MCM1	NP	Q06449	PIN3	NP
P32505	NAB2	NP	P43582	WWM1	NP
Q03761	TAF12	NP	P38996	NAB3	NP
P23291	YCK1	NP	P29295	HRR25	NP
Q12124	MED2	NP	P32896	PDC2	NP
P38080	AKL1	NP	Q02792	RAT1	NP
P25339	PUF4	NP	P32790	SLA1	NP
P39081	PCF11	NP	P22579	SIN3	NP
P43572	EPL1	NP	Q12151	UPC2	NP
P22082	SNF2	NP	P39936	TIF4632	NP
P45978	SCD6	NP	P48562	CLA4	NP
P14680	YAK1	NP	Q06315	SKG3	NP
P53829	CAF40	NP	P39935	TIF4631	NP
P53617	NRD1	NP			

1. Sabate, R., Rousseau, F., Schymkowitz, J. and Ventura, S. (2015) What makes a protein sequence a prion? *PLoS computational biology*, **11**, e1004013.

2. Alberti, S., Halfmann, R., King, O., Kapila, A. and Lindquist, S. (2009) A systematic survey identifies prions and illuminates sequence features of prionogenic proteins. *Cell*, **137**, 146-158.

Table S2. Q/N rich PRLDs-containing proteins in the *S. cerevisiae proteome*, according to PrionW.

Gene name	UniprotKB ID	Uniprot PrD	Amyloid_Core	Prion Domain Q/N>= 25%	Prion Domain Q/N>= 32%	Experimental Prion*
**ERF3**	P05453	5-135	98-RGNYKNFNYNNNLQGYQAGFQ-119	1-254	1-149	**Yes**
**URE2**	P23202	2 – 89	19-NIGNRNSNTTTDQSNINFEFS-40	1-104	1-104	**Yes**
**NEW1**	Q08972	1--153	72-NYNNYNNYNNYNNYNNYNNYN-93	16-119	16-119	**Yes**
**MOT3**	P54785	98 – 295	123-NSNNSNISASDYTVANNSTSN-144	68-177	68-177	**Yes**
**SWI1**	P09547	1 – 323	239-FNNSASNNGNLTSNQLISNYA-260	227-419	227-419	**Yes**
**RNQ1**	P25367	153-402	217-SNNNQNSNNSQQGYNQSYQNG-238	202-405	202-405	**Yes**
**SFP1**	P32432	230 – 458	413-NSNNNSGININNNTSHNSNIN-434	390-463	389-459	**Yes**
**GLN3**	P18494	-	222-NSSSSAMNITNNNNSNNSNIQ-243	179-264	-	Yes
**KSP1**	P38691	-	569-NFNNGNSYIKGWNKNFNKYRR-590	495-625	-	Yes
**LSM4**	P40070	-	139-QNRQYNNSNSSNINNSINSIN-160	77-187	-	Yes
**NRP1**	P32770	-	495-NNNNNINNMTNNRYNINNNIN-516	472-584	-	Yes
**ASM4**	Q05166	-	69-VNANNYSSNIGNNSINNNNIK-90	5-194	-	Yes
**YBL081W**	P38180	-	165-NNSNNNNNLYNQTQFSTRYFN-196	23-206	-	Yes
**MED2**	Q12124	-	267-GNNYNDINISSIENNINNNIN-288	237-431	-	No
**PUF4**	P25339	-	517-HGYYNNNNNNNNNNNNNNNSN-538	451-562	-	No
**AFT1**	P22149	-	527-HNEYILQYLTHSDAANHNNIG-548	493-553	-	-
**AIR2**	Q12476	-	313-TYVDNNSISNSSNYRNYNSYQ-334	268-341	-	-
**COY1**	P34237	-	231-RIAEYNLVTQELETTQARIYQ-252	192-252	-	-
**CDC27**	P38042	-	371-NNNNNNNNNNNNNNNNNNIIN-392	335-411	-	-
**CLG1**	P35190	-	340-SYQSNSRSEFSSMNGYYNYYN-361	332-429	-	-
**DAL81**	P21657	-	35-NSNNNSNHDILNFNDNYTTIL-56	1-162	-	-
**DCP2**	P53550	-	881-LNDGYENISNKDSSHELLNIL-902	880-958	-	-
**FAB1**	P34756	-	468-NNSTITINNLNNTTSNNSNYN-489	430-518	-	-
**GPR1**	Q12361	-	528-NNNNNNNNNNNNNNNNSNNIK-549	446-613	-	-
**HOS1**	Q12214	-	121-NLYNYYLNHSQALENNMDCIN-142	83-142	-	-
**HOT1**	Q03213	-	288-DVNTNTAQLNNQFSNALNTIL-309	265-411	-	-
**HRP1**	Q99383	-	335-GGNNMNRRGGNFGNQGDFNQM-356	296-534	-	-
**HSF1**	P10961	-	501-NDIINDIIFNTNLANNLSNYN-522	440-560	-	-
**MAD1**	P40957	-	290-KLSQLHVLESQYENLQLENID-311	278-406	-	-
**MBI4**	P03879	-	301-QLNNFVLKFNWTKQYNKMNIV-322	301-363	-	-
**MIT1**	P40002	-	351-NNNNNNNNNNNNNNNNINNIN-372	297-465	-	-
**MLP1**	Q02455	-	1024-KIQNDLDQQTIYANTAQNNYE-1045	1004-1064	-	-
**MRN1**	Q08925	-	1-MVVSYNNNNNNNNNNNNNNIS-21	1-167	-	-
**MSN2**	P33748	-	235-SNSISNSNSNSTGNLNSSYFN-256	150-277	-	-
**MSS11**	Q03825	-	413-FQQQALQQNSLQQNLGNQNYQ-434	360-493	-	-
**NAM7**	P30771	-	900-VDNTELSSYINNEYWNFENFK-921	900-961	-	-
**NGR1**	P32831	-	496-DGNFSMEQMAHNNYYNYNNYD-517	459-580	-	-
**NPR3**	P38742	-	703-TNNNIYRFGNNINSTGHCGAA-724	698-757	-	-
**NUD1**	P32336	-	224-SNNKNNNNNNNNNNNNSININ-245	196-292	-	-
**PDR1**	P12383	-	1007-NEINNNNNNNNNNKNNINNIN-1028	982-1057	-	-
**PHO81**	P17442	-	235-NNNNNNNNNNNNNNILHNNYE-256	194-280	-	-
**PIN2**	Q12057	-	232-GENYYYDNNNINNNLQGNSYN-253	184-282	-	-

**PRP9**	P19736	-	74-LQQHEINIFLRDYQEKQQTFN-95	70-130	-	-
**REC8**	Q12188	-	485-EDGHYIEENSQGNILDFNLNL-506	458-517	-	-
**REG2**	P38232	-	78-KNFDFHEQRSLILNLNLWKFI-99	73-152	-	-
**RMAR**	P02381	-	351-TFNNKKYLWSNINNNYKLNYI-372	335-398	-	-
**ROD1**	Q02805	-	511-SASSSAVESQAIHNIQNLYIS-532	507-583	-	-
**RPI1**	P23250	-	195-MQYLLVQLQNTFSFVNGNIIL-216	130-283	-	-
**RPN4**	Q03465	-	152-TFNYDVKISNDFFDNGDNLYG-173	151-210	-	-
**RSC58**	Q07979	-	375-ANSESTENKEQFIIKLQNLYN-396	317-404	-	-
**SLF1**	Q12034	-	236-YKQLSYFRQQQYYNNINYQQQ-257	72-281	-	-
**SLK19**	Q08581	-	402-DASITEVNHIKGEHENTVNTL-423	388-450	-	-
**SLM1**	P40485	-	62-QQSASFQNGSLTSDINQQSYL-83	21-86	-	-
**SPT10**	P35208	-	619-VTKIVNNESNTFTEHNSNIYY-640	567-640	-	-
**SPT20**	P50875	-	91-KQQALQNYEAQFYQMLMTLNK-112	41-116	-	-
**SPT21**	P35209	-	315-QHQNIAYEINTLQNDNTIQRT-336	291-353	-	-
**SWI4**	P25302	-	232-DNHTTMNFNNDTRHNLINNIS-253	141-476	-	-
**TDA9**	Q04545	-	422-NESIEKSQNNSNVINETIDHT-443	384-454	-	-
**VIK1**	Q12045	-	176-QESQELYNNKLIFWENELQIM-197	175-143	-	-
**VPS60**	Q03390	-	28-QQSLSNRISQLDTQIAQLNFQ-49	1-104	-	-
**YML053C**	Q04978	-	72-GCANLNNNNNNNIIINNNNNNN-93	1-164	-	-
**ZDS2**	P54786	-	227-YLELIQDTLQNIQISTNQDID-248	208-268	-	-
**ZRG8**	P40021	-	960-KIELTNKTVTFFNSNNWNTYD-981	958-1018	-	-

*Actual prions are shown in bold.

Table S3. Recovery of proteins from Table S1 by DIANA when used to analyse the *S. cerevisiae* proteome.

Uniprot Entry	Gene names	TP/TN/FP/FN*	Uniprot Entry	Gene names	TP/TN/FP/FN*
P38691	KSP1	TP	P50896	PSP1	FP
Q12221	PUF2	TP	P25339	PUF4	FP
P25367	RNQ1	TP	Q00772	SLT2	FP
P18480	SNF5	TP	P22082	SNF2	FP
P23202	URE2	TP	Q03761	TAF12	FP
P38180	YBL081W	TP	Q12151	UPC2	FP
Q08972	NEW1	TP	P14680	YAK1	FP
P32770	NRP1	TP	Q06251	YLR177W	FP
Q05166	ASM4	TP	P39523	EPO1	FP
P40070	LSM4	TP	P53617	NRD1	TN
P05453	SUP35	TP	Q02792	RAT1	TN
P38080	AKL1	FP	P32790	SLA1	TN
P41696	AZF1	FP	P22579	SIN3	TN
P31384	CCR4	FP	P48562	CLA4	TN
Q05785	ENT2	FP	Q06315	SKG3	TN
P43572	EPL1	FP	P39935	TIF4631	TN
P29295	HRR25	FP	P24276	SSD1	TN
P39936	TIF4632	FP	Q08831	VTS1	TN
P23291	YCK1	FP	P53309	YAP1802	TN
P11746	MCM1	FP	P32900	SKG6	TN
Q12124	MED2	FP	Q06449	PIN3	TN
P32505	NAB2	FP	P43582	WWM1	TN
P38996	NAB3	FP	P45978	SCD6	TN
P48837	NUP57	FP	P53829	CAF40	TN
P39081	PCF11	FP	P18494	GLN3	FN
P32896	PDC2	FP			

*TP = True Positives, FP = False Positives, TN = True Negatives and FN = False Negatives.

Table S4. Proteins identified by both PrionW and DIANA in the *S.cerevisiae* proteome.

Gene names	Uniprot Entry	Experimental Prion*
ERF3	P05453	**Yes**
RNQ1	P25367	**Yes**
SWI1	P09547	**Yes**
URE2	P23202	**Yes**
NEW1	Q08972	**Yes**
KSP1	P38691	Yes
LSM4	P40070	Yes
NRP1	P32770	Yes
ASM4	Q05166	Yes
YBL081W	P38180	Yes
MOT3	P54785	**Yes**
SFP1	P32432	**Yes**
MED2	Q12124	No
PUF4	P25339	No
CDC27	P38042	-
DAL81	P21657	-
FAB1	P34756	-
GPR1	Q12361	-
HOT1	Q03213	-
MAD1	P40957	-
MIT1	P40002	-
MSS11	Q03825	-
NGR1	P32831	-
PDR1	P12383	-
PHO81	P17442	-
RMAR	P02381	-
RPI1	P23250	-
SLF1	Q12034	-
SLM1	P40485	-
SPT20	P50875	-
SWI4	P25302	-
YML053C	Q04978	-

*Actual prions are shown in bold.

Table S5. Recovery of proteins from Table S1 by LPS when used to analyse the *S. cerevisiae* proteome.

Uniprot Entry	Gene name	TP/TN/FP/FN	Uniprot Entry	Gene name	TP/TN/FP/FN
Q12221	PUF2	TP	P22579	SIN3	FP
P09547	SWI1	TP	Q12151	UPC2	FP
P38691	KSP1	TP	P48562	CLA4	FP
Q05166	ASM4	TP	Q00772	SLT2	FP
P23202	URE2	TP	P41696	AZF1	FP
P18494	GLN3	TP	P31384	CCR4	FP
P25367	RNQ1	TP	P48837	NUP57	FP
Q08972	NEW1	TP	P24276	SSD1	FP
P32770	NRP1	TP	Q08831	VTS1	FP
P38180	YBL081W	TP	P50896	PSP1	FP
P05453	SUP35	TP	P53309	YAP1802	FP
P11746	MCM1	FP	P39523	EPO1	FP
P32505	NAB2	FP	Q05785	ENT2	FP
Q03761	TAF12	FP	P32900	SKG6	FP
P23291	YCK1	FP	Q06251	YLR177W	FP
Q12124	MED2	FP	Q06449	PIN3	FP
P38080	AKL1	FP	P43582	WWM1	FP
P25339	PUF4	FP	P38996	NAB3	FP
P39081	PCF11	FP	P40070	LSM4	FP
P43572	EPL1	FP	P39935	TIF4631	TN
P22082	SNF2	FP	P45978	SCD6	TN
P14680	YAK1	FP	P53617	NRD1	TN
P53829	CAF40	FP	Q02792	RAT1	TN
P32896	PDC2	FP	P39936	TIF4632	TN
P32790	SLA1	FP	Q06315	SKG3	TN
P29295	HRR25	FP			

*TP = True Positives, FP = False Positives, TN = True Negatives and FN = False Negatives.

Table S6. Proteins identified by both PrionW and LPS in the *S.cerevisiae* proteome.

Gene names	Uniprot Entry	Experimental Prion*
ERF3	P05453	**Yes**
RNQ1	P25367	**Yes**
SWI1	P09547	**Yes**
URE2	P23202	**Yes**
NEW1	Q08972	**Yes**
GLN3	P18494	Yes
KSP1	P38691	Yes
LSM4	P40070	Yes
NRP1	P32770	Yes
ASM4	Q05166	Yes
YBL081W	P38180	Yes
MOT3	P54785	**Yes**
SFP1	P32432	**Yes**
MED2	Q12124	No
PUF4	P25339	No
CDC27	P38042	-
DAL81	P21657	-
FAB1	P34756	-
GPR1	Q12361	-
HOT1	Q03213	-
HSF1	P10961	-
MAD1	P40957	-
MIT1	P40002	-
MRN1	Q08925	-
MSN2	P33748	-
MSS11	Q03825	-
NGR1	P32831	-
NUD1	P32336	-
PDR1	P12383	-
PHO81	P17442	-
RPI1	P23250	-
SLM1	P40485	-
SWI4	P25302	-
YML053C	Q04978	-

*Actual prions are shown in bold.

Table S7. Amyloid core and pWALTZ score of Q/N rich PrLDs in human prion-like proteins linked to disease. Proteins were identified by PrionW using Q/N content ≥ 15 % and pWALTZ cut-off = 64.00.

Uniprot Entry	Protein name	Amyloid Core	PrionW Score
P09651	hnRNPA1	SGSNFGGGGSYNDFGNYNNQS	64.95
Q99729	hnRNPA2	GQSQSWNQGYGNYWNQGYGYQ	76.00
P51991	hnRNPA3	DGYNEGGNFGGGNYGGGGNYN	65.28
O14979	hnRNPDL	QGQNWNQGFNNYYDQGYGNYN	82.27
P35637	FUS	QDNSDNNTIFVQGLGENVTIE	69.33
Q01844	EWS	ASTDYSTYSQAAAQQGYSAYT	69.77
Q92804	TAF15	ASQSYSGYGQTTDSSYGQNYS	69.82
O75177	SS18L1/CREST	SSQQYLGQEEYYGEQYSHSQG	70.81
